# Investigation of a prolonged nursery outbreak of *Salmonella* Poona in England identified using whole genome sequencing, 2016–2021

**DOI:** 10.1017/S0950268826101393

**Published:** 2026-04-07

**Authors:** Joanna Garner, Caisey Pulford, Valérie Decraene, Iona Smith, Vicky Watts, Anaïs Painset, Marie Anne Chattaway, Anna Trelfa

**Affiliations:** 1 Field Service North West, UK Health Security Agency, UK; 2 UK Field Epidemiology Training Programme, UK Health Security Agency, UK; 3 Gastrointestinal Pathogens and Food Safety (One Health) Division, UK Health Security Agency, UK; 4 NIHR Health Protection Research Unit in Gastrointestinal Infections, Liverpool, UK; 5 Gastrointestinal Bacterial Reference Unit, Public Health Microbiology Division, UK Health Security Agency – Colindale, UK; 6 NIHR Health Protection Research Unit in Public Health Genomics, UK; 7 North West Health Protection Team, UK Health Security Agency North West, UK

**Keywords:** carriage, cholecystectomy, outbreak, *S. Poona*, *Salmonella enterica*, whole genome sequencing

## Abstract

We describe a prolonged outbreak of *Salmonella enterica* serotype Poona (*S.* Poona) sequence type (ST) 308, which comprised 13 cases occurring intermittently in North West England between 2016 and 2021. Whole genome sequencing (WGS) results indicated potential exposure to a single source but a lack of good quality data from routine surveillance questionnaires initially made it challenging to identify the cause. Continuing identification of cases in young children in a small geographical area prompted further public health actions, including trawling interviews which identified that ten cases attended the same nursery. As part of enhanced case finding in this nursery, childcare staff were asked to submit faecal samples. One asymptomatic staff member was positive for *S.* Poona and had worked at another nursery, attended at the time by the first *S.* Poona child case in this outbreak. Further investigations revealed that the case had previously undergone a cholecystectomy. We report an outbreak caused by persistent carriage and shedding of *S.* Poona in an asymptomatic individual working with vulnerable groups, which necessitated introduction of risk management measures similar to that for Typhoidal *Salmonella.* We also demonstrate the utility of combining epidemiological and WGS data in the public health response to *Salmonella* outbreaks.

## Introduction

*Salmonella* Poona (*S.* Poona) is a serotype of non-typhoidal *Salmonella* bacteria, which typically causes gastroenteritis symptoms including fever, abdominal pain, diarrhoea, nausea, and vomiting. Infections do not usually require treatment although children and elderly people are particularly at risk of severe dehydration and invasive infection [1]. Transmission of the bacteria occurs through consumption of contaminated food, person-to-person spread through the faecal-oral route, and also through contact with infected animals. Children under 5 years old are particularly susceptible to acquiring and transmitting gastrointestinal infections, and children are also at greater risk of developing a more severe clinical illness from *Salmonella* [2].

Across the European Union/European Economic Area (EU/EEA) countries, 147–206 *S.* Poona cases were reported between 2013 and 2017 [3]. The Poona serotype is rarely detected in England, typically comprising approximately 0.5% of about 9,000 *Salmonella* cases annually [4, 5] (average of 45 infections in a year, UKHSA data, unpublished).

Several outbreaks of *S.* Poona related to food and drink consumption have been reported across Europe. For example, a large national outbreak of 285 confirmed cases of *S.* Poona detected over an 18-month period was investigated in Spain in 2011 and found to be associated with consumption of infant formula milk [6]. A further outbreak investigation in France in 2018 identified 31 infant cases of *S.* Poona, which were found to be associated with rice-based infant formula [7]. Three EU countries reported food-borne outbreaks to the European Food Standards Agency (EFSA) between 2009 and 2017; 26 cases in Hungary were suspected to be associated with consumption of a pig meat product although the investigation of two further outbreaks in Austria and Sweden did not find strong evidence of an association with a specific food product [3]. In the United States, notable outbreaks have been linked to exposure to pet turtles, contaminated cantaloupe melon, and cucumbers [8–10].

## Outbreak detection

In January 2021, a cluster of four genetically linked cases of *S.* Poona were identified through routine WGS-based surveillance with specimen dates from 2016 (two cases), 2018, and 2020. The cases were resident in the same city borough in North West England, and due to the exceedance of expected cases of this rare serotype, the health protection team investigated. No epidemiological link between cases was identified from the routine surveillance questionnaires available. Given the extended time period over which the cases occurred, it was considered that no further investigations were necessary at this stage.

Between January and October 2021, a further eight cases were genetically linked to this cluster with specimen dates in 2018 (one case), 2020 (one case), and 2021 (six cases) in the same city borough in North West England. Upon review of all available routine surveillance questionnaires, it was noted that 10 of the 12 cases were aged under 2 years at symptom onset. Nursery attendance was only indicated for two of these cases, but both attended the same nursery.

In October 2021, following identification of the nursery setting, an outbreak control team (OCT) was convened to investigate the 12 genetically linked cases identified. Here, we describe the investigation of this prolonged outbreak caused by *S.* Poona and share our learning.

## Methods

### Human microbiological investigation

The *Salmonella* reference microbiology service at Public Health England [now UK Health Security Agency (UKHSA)] established the use of WGS for routine *Salmonella* surveillance in 2015, providing the means for detailed strain characterization, typing, and detection of clusters of genetically similar isolates [11]. These isolates are grouped using a single linkage clustering (SLC) approach based upon single nucleotide polymorphism (SNP) differences, generally with a 5-SNP threshold being used for outbreak detection [12].

### Case definition

The confirmed case definition was a laboratory-confirmed case of *S.* Poona belonging to the same 5-SNP SLC as another confirmed outbreak strain according to the UKHSA’s SNP pipeline. A probable case was defined as any *Salmonella* infection confirmed by culture or polymerase chain reaction (PCR) where WGS results were awaited or unavailable in someone with an epidemiological link to the nursery of concern.b

### Routine surveillance

Confirmed cases of non-typhoidal *Salmonella* are sent a routine electronic surveillance questionnaire by the local health protection team for self-completion. The questionnaire asks about the nature, onset, and severity of symptoms; illness in household contacts; educational and workplace settings; travel history; foods consumed outside the home; and animal contact. Response rates and questionnaire completion can vary, so case interviews are often required to support hypothesis generation and testing.

### Epidemiological investigation

The data available in the surveillance questionnaires and the case management records were summarized in terms of cases over time, broken down by age, sex, and common exposures. For hypothesis generation, a trawling questionnaire was designed to identify common exposures among the cases. The trawling questionnaire was used to confirm data items collected in the routine surveillance questionnaire, as well as to ask additional questions. Information was gathered regarding the nature, severity, and onset of symptoms; comorbidities; symptoms in household contacts; travel history; contact with animals; food exposures including children’s snacks; consumption of formula milk; and eating out and where food was purchased. Trained interviewers completed questionnaires over the telephone with a parent/guardian of six child cases with onset in 2021. Information was only gathered from the most recent cases to avoid the likely recall bias if earlier cases were interviewed.

### Environmental investigations

Local authority environmental health officers conducted a site visit at the nursery to visually identify potential hazards within the setting and to collect environmental samples.

### Enhanced case finding

Once an outbreak had been declared, the OCT continued to monitor WGS results to identify additional cases falling within the same 5-SNP SLC. The sickness absence record at the nursery was reviewed to determine whether there were children or staff with recent gastrointestinal illness and therefore could be possible cases. All staff with childcare responsibilities in the nursery room most affected were asked to submit faecal samples for testing to investigate whether there was a chronic carrier of *S.* Poona in the setting. There was scope to widen the sampling strategy to all staff members if required.

### Phylogenetic analysis

To determine the genetic relationship between cases, a maximum likelihood phylogenetic tree was constructed. Isolates were sequenced on an Illumina sequencing platform [13, 14] and Illumina reads were mapped to the *S.* Poona reference genome (NCTC4840) using BWA v0.7.12 and Samtools v1.1 [15, 16]. High-quality variants (SNPs) were identified using GATK v2.6.5 in 160 unified genotyper mode [17]. High-quality core-genome SNPs (>90% consensus, minimum depth 161 10×, mapping quality ≥30) were extracted from SnapperDB v0.2.8 [12]. Gubbins v2.0.0 [18] was used to mask horizontally acquired SNPs from the alignment before phylogeny generation. IQtree v2.0.4 [19] was used to derive the maximum likelihood phylogeny of the genomes. Visualization and annotation of the phylogeny were performed through the iTol platform. Sequences were analysed for quality control, speciation, sequence type, and serotype as previously described [13].

## Results

### Description of cases detected in outbreak

**Figure 1.**
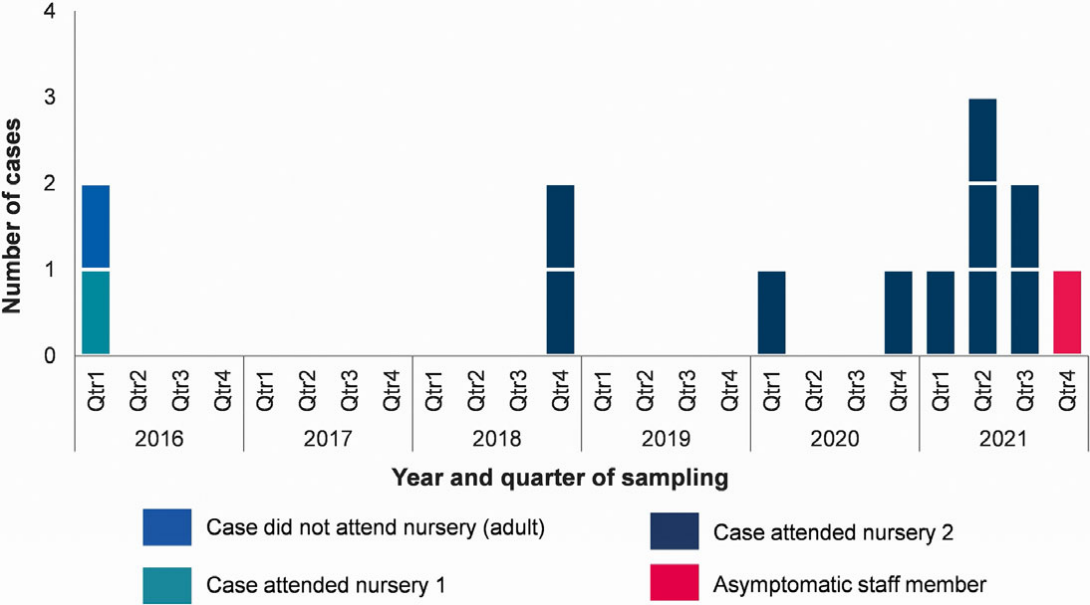
Confirmed cases of *Salmonella* Poona belonging to outbreak by setting, North West England, 2016–2021 (*n* = 13*). *12 cases were reported via routine surveillance; the final case was identified via staff sampling.

As of 21 October 2021, there were 12 confirmed cases with sample dates from 16 March 2016 to 24 August 2021 (Figure 1). Descriptive epidemiological investigations demonstrated a fairly even distribution of cases between males and females (7/12, 58% were male). The majority of cases (11/12; 92%) were children aged under 2 years. The remaining case was an adult, with a sample date of March 2016 who reported international travel prior to symptom onset. Information on hospitalisation was available for 11 cases, 5 of whom (45%) reported being hospitalized.

### Epidemiological investigation

The trawling questionnaire did not elicit many commonalities between cases (Supplementary Table S1). Upon review of the original questionnaires coupled with the trawling questionnaires, all 11 child cases were found to attend nurseries. The earliest of these cases (sample date March 2016) had attended Nursery 1, while the remaining ten cases (sample dates October 2018–August 2021) had attended Nursery 2 at the time of their infection. Notably, 5/6 parents/guardians interviewed for the trawling questionnaire perceived the illness may have originated from the Nursery 2 setting.

### Environmental investigations

None of the environmental samples at Nursery 2 tested positive for *S.* Poona. In a section of the nursery for children mostly aged 1–2 years old, 10/22 samples tested positive for Enterobacteriaceae, indicating a need for hygiene improvements in this area. The nappy changing and disposal areas were visibly clean, but the handwashing basin used after nappy changes was also used by children and staff for washing after play activities.

### Enhanced case finding

Staff sampling led to the identification of an asymptomatic case of the outbreak strain at Nursery 2. The staff member had also worked at Nursery 1 in 2016, which was attended at the time by the first *S.* Poona child case in this outbreak. Upon clinical review, the staff case reported being asymptomatic over the past six years, and they reported having undergone a cholecystectomy.

No additional cases were identified from reviewing sickness absence records at Nursery 2 for children or staff who had had recent gastrointestinal illness.

### Phylogenetic analysis

**Figure 2.**
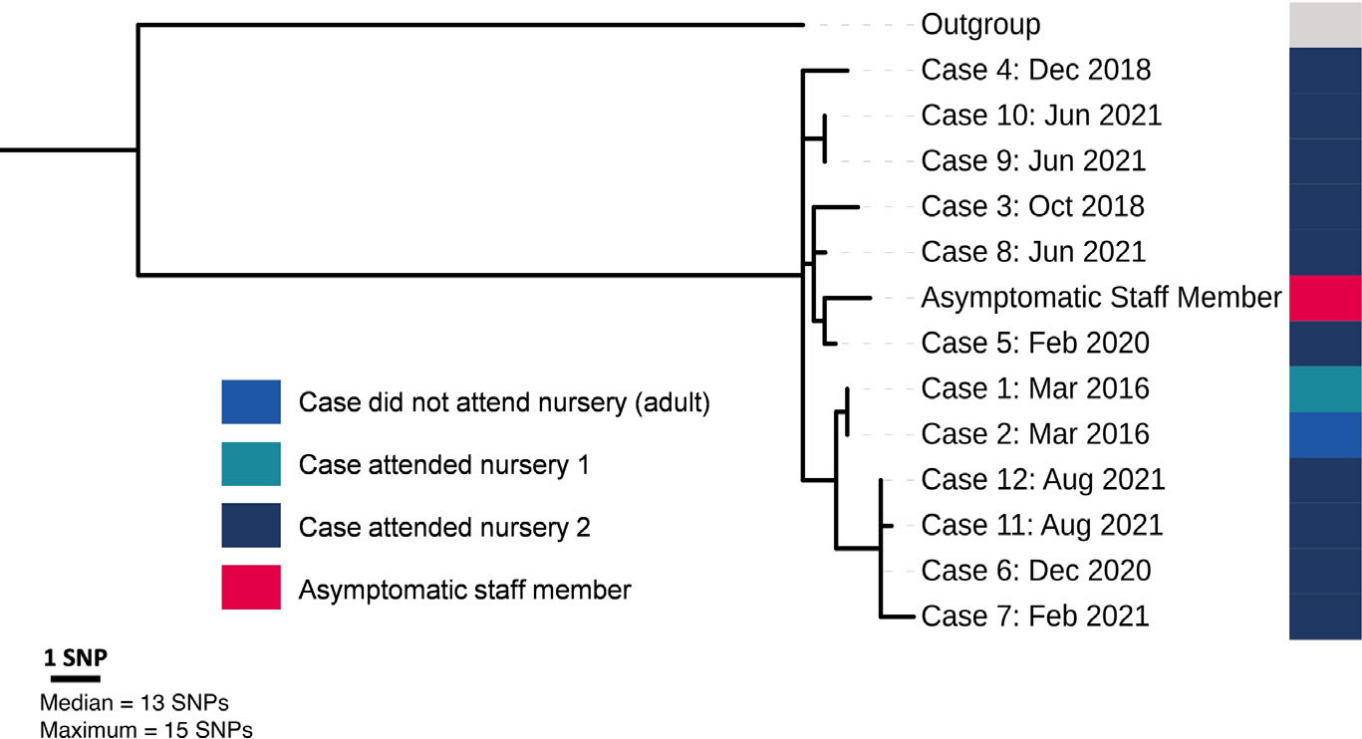
Maximum likelihood phylogenetic tree demonstrating population structure of all laboratory-confirmed cases of *Salmonella* Poona belonging to the same 5-SNP* Single linkage clustering as another confirmed outbreak strain according to UKHSA’s SNP* pipeline (*n* = 13). *SNP = Single nucleotide polymorphism.

Despite the outbreak spanning six years, the phylogenetic analysis (Figure 2) demonstrated a reasonably close genetic relationship between all 13 cases, including the asymptomatic individual identified through screening. The median and maximum pairwise distance was 12.5 and 15 SNPs respectively, and temporal clustering of isolates was evident. The isolate sampled from the asymptomatic staff member was phylogenetically situated among isolates sampled from symptomatic cases and was therefore within the outbreak cluster.

### Outbreak control measures

Infection control practices were reinforced in the nursery setting while the outbreak was being investigated. This included education on good hand hygiene practices, exclusion policies (enforcing 48-h exclusion from the nursery after episodes of vomiting or diarrhoea), cleaning policies, and self-audit to encourage compliance with the control measures.

A communications plan was created to increase awareness of symptoms, signposting to primary care and enteric precautions. Letters were provided to staff to explain the rationale for sampling and the setting was given written advice by infection control nurses and environmental health officers, which was verbally reiterated during site visits. A newsletter distributed to all nurseries in the borough included a piece on awareness of gastrointestinal illness.

The staff member who tested positive for *S.* Poona was clinically reviewed by an infectious diseases consultant and prescribed antibiotic treatment. A clearance sampling schedule was agreed: three negative stool cultures at 48-h intervals 1 week after antibiotic treatment had finished. The OCT initially advised that the case be excluded from work due to working with a vulnerable age group. This advice was updated after an evidence review; the risk of transmission was determined to be low if the case was redeployed to an administrative role, was asymptomatic, had their own toilet and handwashing facilities, and the Community Infection Control Team provided enhanced infection prevention and control support to the nursery. After several weeks of antibiotic treatment, the case cleared the infection.

Surveillance data for the area was actively monitored for a 6-month period. If any new cases of *S.* Poona were identified, the health protection team planned to actively follow them up (rather than asking cases to self-complete questionnaires). No new *S.* Poona cases were detected in this time frame and after the 6-month period ended, routine local and national surveillance processes were resumed.

## Discussion

This investigation demonstrates the utility and importance of combining epidemiological and WGS data to identify and investigate intermittent *Salmonella* outbreaks. Based on clinical and epidemiological assessment, it is likely that the staff member case had persistent carriage of the outbreak strain over at least six years and was the source of infection in both nurseries.

Given the long time frame, these cases would have likely been classified as ‘sporadic’ and not investigated as part of a cluster in the absence of integrated WGS and epidemiological data. UKHSA established the use of WGS as part of routine *Salmonella* surveillance in 2015. Since its implementation, this approach has facilitated the detection of multiple local outbreak clusters and has additionally facilitated the consolidation of multiple regional outbreaks into single, larger, national level outbreaks. The integration of WGS into routine *Salmonella* surveillance has subsequently been adopted internationally, facilitating detection of multicountry outbreaks and assisting with coordinated multicountry epidemiological investigations [20]. The challenge is how to best use this complex additional information to inform and investigate future outbreaks. This needs to build upon the practice of collaborative working between the reference laboratory, epidemiologists and public health specialists, including joint assessment of the current risk to public health, particularly in the context of a historic cluster.

The majority of outbreak investigations have identified local/dispersed food-borne transmission routes (albeit some secondary person–person transmission may have contributed) [4] outbreaks with prolonged person-to-person transmission are rarely described. In other countries (particularly those in sub-Saharan Africa), there is growing evidence for person-to-person transmission of invasive variants of non-typhoidal *Salmonella* serovars (e.g. *Salmonella Typhimurium* ST313) [21]. These variants are thought to have evolved to survive in immunocompromised individuals and have hallmark genomic signatures of invasiveness (primarily extensive pseudogenisation and certain accessory genome components such as prophages).

Interestingly, among those non-typhoidal *Salmonella* serovars listed in the top 30 serovars in England, *S.* Poona has a high proportion of invasive infections (14% of cases have a sample taken from bloodstream; UKHSA data, unpublished). Our findings of human-to-human transmission among *S.* Poona cases are especially noteworthy, warranting additional studies to better understand the invasive properties of this pathogen. The hospitalization rate of 45% in this cluster was higher than that usually reported in salmonellosis outbreaks (21%) [22] and may be influenced by the young age of those affected. It is also a possible indicator of increased clinical severity of infection within the cluster.

Nurseries will be following infection control policies [23] as standard which include guidance on handwashing, personal protective equipment, cleaning, exclusion advice, etc., but it is important to acknowledge that nurseries are not clinical settings, and there are likely to be items such as toys and play equipment that are difficult to keep clean and need careful environmental management. A limitation of the investigation was that we were unable to quantify the full extent of the outbreak; we assume that there was a lapse in infection control measures to allow transmission, but do not know what can be attributed to direct person-to-person spread and to environmental contamination.

This outbreak highlights the possibility of persistent carriage and shedding of *S.* Poona, which is a finding not well documented in the literature for non-typhoidal *Salmonella* [24]. The finding that the staff member case had undergone a cholecystectomy was of note as gallbladder disease has been linked to chronic carriage of *Salmonella Typhi* and possibly non-typhoidal *Salmonella* [24–26]. Our investigations might have been expedited had we known about the staff member case’s underlying medical history sooner and recommend that in future outbreaks a review of occupational health records or asking staff about relevant medical history is done as part of case finding. In this instance, the case did not tolerate antibiotics well, experienced side effects during their treatment, and needed several types of antibiotics before clearing the infection which delayed their return to their normal duties. Conducting a risk assessment for a case slow to clear the infection or a persistent carrier who works with vulnerable groups necessitates consideration of more enhanced risk management measures such as temporary or permanent exclusion of the individual from that setting or changing their duties until clearance is achieved.

Case-finding was difficult in this setting with staff initially being hesitant to submit samples. This is a common challenge in health protection; communicating the need for screening and the potential implications for an individual should they test positive can be a difficult conversation to navigate. In the management of this investigation, it was important to keep the staff-case and their employer engaged; the environmental health officers had a vital liaison role in managing relationships and building rapport with staff to facilitate sample submission and ensuring the control measures were followed. Establishing and maintaining good relationships with settings in order to ensure satisfactory outcomes is key before, during and after outbreaks.

There was no sick pay or financial support available for the case while excluded from work in this situation, presenting a risk of financial hardship. This potential impact for individuals could discourage cases from reporting symptoms and being tested or screened in future situations, adversely impacting the ability to effectively control outbreaks. In early years settings and social care, where workers are often on a low wage, there is a particular risk of transmission to more vulnerable groups such as the very young and the elderly. Research undertaken during the COVID-19 pandemic showed that appropriate financial support for cases can promote outbreak control [27, 28] and in future outbreaks, we recommend that this support is available.

The investigation highlighted the difficulty of relying on self-completed questionnaires at the time of infection for outbreaks over long periods of time. Once the outbreak had been identified, there was an absence of complete intelligence early on from passive surveillance, and from later cases which had occurred during the COVID-19 pandemic, which necessitated in-depth interviews with cases. It was identified through this outbreak that while the self-completed questionnaires did include questions on educational settings with prompts for school and university details, it did not include a question on childcare or nursery settings. A standard question on nursery or childcare settings has now been added to the self-completed non-typhoidal *Salmonella* questionnaire.

We have described a challenging long-running outbreak which demonstrated the benefits and utility of linking genomic and epidemiological data for routine *Salmonella* surveillance. We also reported the unusual finding of persistent asymptomatic carriage with a rare serotype of *Salmonella.* The investigation highlighted data quality issues associated with self-completed surveillance questionnaires and, as a result of this outbreak, the surveillance questionnaire has been improved. Once the control measures were implemented, no further cases were reported, showing the success and appropriateness of the measures taken.

## Supporting information

10.1017/S0950268826101393.sm001 Garner et al. supplementary material Garner et al. supplementary material

## Data Availability

FASTQ sequences were deposited in the NCBI Short Read Archive under the BioProject PRJNA248792 (https://www.ncbi.nlm.nih.gov/bioproject/?term=248792).

## Supplementary material

The supplementary material for this article can be found at http://doi.org/10.1017/S0950268826101393.
