# Supplementary material for: Investigation of a prolonged nursery outbreak of Salmonella Poona in England identified using whole genome sequencing, 2016–2021
Source: Epidemiol Infect. 2026 Apr 7;154:e52. doi: 10.1017/S0950268826101393 (PMC13161793; doi:10.1017/S0950268826101393)
Supplement: Garner et al. supplementary material [file S0950268826101393sup001.docx]

Supplementary Information

**Table S1:** Summary of trawling questionnaire data collected from confirmed cases of *Salmonella* Poona (n=6)

| **Exposure** | **Number of cases (%)** |
| --- | --- |
| **Other medical conditions** | |
| Undergoing treatment for other conditions | 0/6 (0) |
| **Household details** | |
| Close contact with anyone with diarrhoea in the 7 days before onset | 0/6 (0) |
| **Travel details** | |
| Travelled outside the UK within the 7 days before onset | 0/6 (0) |
| Contact with anyone with some who had travelled outside the UK within the 7 days before onset | 0/5 (0),  1 no response |
| **Contact with animals** | |
| Visited an open farm, fed farm animals or ate at the premises | 1/6 (17) |
| Had contact with pets at home | 1/6 (17) |
| **Activities and outings** | |
| Visited a local park | 2/6 (33) |
| **Diet** | |
| Halal | 1/6 (17) |
| Vegetarian | 1/6 (17) |
| Lactose-Free | 1/6 (17) |
| Egg-free | 1/6 (17) |
| **Infant food exposures** | |
| Brand A products | 4/6 (67) |
| Brand B products | 2/6 (33) |
| **General food exposures** | |
| At a kebab shop | 1/6 (17) |
| Omelettes/scrambled/hard boiled eggs (Lion marked eggs) | 3/6 (50) |
| Takeaway pizza | 1/6 (17) |
| **Additional information** | |
| Suspected the illness may have been acquired at nursery | 5/6 (83) |
| Cases provided a food history from the nursery setting using the nursery app | 1/6 (17) |
